# Efficacy and safety of behavioral therapy for premature ejaculation: Protocol for a systematic review

**DOI:** 10.1097/MD.0000000000014056

**Published:** 2019-01-18

**Authors:** Binghao Bao, Jianwei Shang, Jisheng Wang, Hengheng Dai, Xiao Li, Kaige Zhang, Haisong Li, Bin Wang

**Affiliations:** a Graduate School of Beijing University of Chinese Medicine; b Department of Andrology, Dongzhimen Hospital, Beijing University of Chinese Medicine; c Department of Encephalopathy, Dongzhimen Hospital, Beijing, China.

**Keywords:** behavioral therapy, premature ejaculation, protocol, systematic review

## Abstract

**Background:**

Premature ejaculation (PE) is the one of the most prevalent male sexual dysfunction, there has not been specific medicine or therapy for the disease. As an effective treatment for premature ejaculation is a behavioral therapy and is widely used worldwide. The purpose of this study is to evaluate the efficacy and safety of behavioral therapy in patients who suffer from PE.

**Method:**

We will search all randomized controlled trials (RCTs) from the following electronic databases, by September 30, 2018, such as PubMed, EMBASE, the Cochrane Library, Web of Science database, China Biology Medicine disc (CBM), China National Knowledge Infrastructure (CNKI), China Science and Technology Journal database (VIP), and Wanfang Database. We will also collect clinical trial registries, dissertations, grey literature, reference lists of studies, systematic reviews, and conference abstracts. The primary outcomes include the Intravaginal Ejaculatory Latency Time (IELT). Besides, Premature Ejaculation Diagnostic Tool (PEDT), Arabic index of Premature Ejaculation (AIPE), Index of Premature Ejaculation (IPE) will be the secondary outcomes. Two people will review these articles, extract the data information, and assess the quality of studies separately. RevMan (version 5.3) and EndNote X7 will be used for meta-analysis.

**Results:**

This study will generate a comprehensive review of current evidence of behavioral therapy for premature ejaculation.

**Conclusion:**

The study will provide updated evidence to evaluate the efficacy and safety of behavioral therapy for premature ejaculation.

**Ethics and dissemination:**

It is not necessary for this systematic review to acquire an ethical approval. This review will be reported in a peer-reviewed journal.

**PROSPERO registration number:**

PROSPERO CRD42018111339.

## 1 Introduction

Premature ejaculation (PE) is one of the most common male sexual dysfunction diseases, which may have adversely affected 20% to 30% of the male population.^[1–2]^ Premature ejaculation has a serious negative impact on the sexual life satisfaction of patients and their sexual partners.^[3]^ The prevalence of PE did not vary significantly in young and middle-ages men, indicating that no particular age group has consistently been shown to be at greater risk for PE.^[4–5]^ According to the International Society for Sexual Medicine (ISSM),^[6]^ PE is a male sexual dysfunction which has the following symptoms: Ejaculation that always or nearly always occurs before or within about 1 minute of vaginal penetration (lifelong PE) or a clinically significant and bothersome reduction in latency time, often to about 3 minutes or less (acquired PE), accompanied by the inability to delay ejaculation on all or nearly all vaginal penetrations and negative personal consequences, such as distress, dysphoria, frustration, and/or the avoidance of sexual intimacy. Besides lifelong PE and acquired PE, 2 more types of PE have been proposed: natural variable PE and premature-like ejaculatory dysfunction.^[7–9]^

The treatment of premature ejaculation mainly includes drug therapy and psychological/behavioral therapy. Selective serotonin reuptake inhibitors (SSRIs) are acknowledged effective drugs for the treatment of PE, including fluoxetine, paroxetine, sertraline, dapoxetine, and so on.^[10–13]^ Dapoxetine hydrochloride is a short-acting SSRI and it is the only drug approved for on-demand treatment of PE in European countries and elsewhere.^[13]^

In a prospective randomized trial, the combination of dupoxetine with behavioral therapy was more effective in treating lifelong PE patients than the use of dupoxetine alone.^[14–15]^ Therefore, it is necessary to conduct a meta-analysis on premature ejaculation to evaluate the efficacy and safety of behavioral therapy. In this review, we aim to assess the efficacy and safety of behavioral therapy for treating premature ejaculation.

## 2 Methods

### 2.1 Study registration

The protocol of review methods has been registered, prospectively (CRD42018111339; https://www.crd.york.ac.uk/PROSPERO/display_record.php?RecordID=111339). The protocol has obeyed from Preferred Reporting Items for Systematic Reviews and Meta-Analyses Protocols (PRISMA-P) statement guidelines. We will document the essential protocol amendments in the full review.

### 2.2 Inclusion criteria for study selection

#### 2.2.1 Types of studies

The type of literature included will be randomized controlled trials (RCTs) of behavioral therapy for PE. The language is limited to Chinese and English. Non-RCTs, quasi-RCTs, case series, case reports, and crossover studies will be excluded.

#### 2.2.2 Types of patients

The cases included are adult male patients over 18 years old who have diagnosed PE. The region, nation, ethnic, and sources are not limited.

#### 2.2.3 Types of interventions

##### 2.2.3.1 Experimental interventions

Behavioral therapy as experimental groups can be included, such as ‘stop-start’, ‘squeeze’, ‘sensate focus’, and pelvic floor muscle rehabilitation. The treatment duration and frequency are not limited.

##### 2.2.3.2 Control interventions

The control groups can be using no treatment, sham treatment and placebo. The following treatment comparisons will be investigated:

- behavioral therapy versus no treatments;
- behavioral therapy versus placebo or sham treatment;
- behavioral therapy versus other treatments;
- behavioral therapy adjunctive to other treatments versus other treatments alone;
- behavioral therapy adjunctive to other treatments versus placebo or sham treatment adjunctive to other treatments.

#### 2.2.4 Types of outcome measures

##### 2.2.4.1 Primary outcomes

The primary outcome measurement will be the Intravaginal Ejaculatory Latency Time (IELT).

##### 2.2.4.2 Secondary outcomes

1. Premature Ejaculation Diagnostic Tool (PEDT);
2. Arabic index of Premature Ejaculation (AIPE);
3. Index of Premature Ejaculation (IPE);
4. Sexual satisfaction.
5. Adverse events.

### 2.3 Search methods for the identification of studies

#### 2.3.1 Electronics searches.

The literature research will be divided into electronic search and manual search, by the time of September 30, 2018. Electronic search databases include Pubmed, EMBASE, The Cochrane Library, the Chinese BioMedical Literature Database, the China National Knowledge Infrastructure (CNKI), the China Science and Technology Journal database (VIP), and the Wanfang database. For a more complete search of the database, the team members will develop an elaborate search strategy based on the Cochrane Handbook Guidelines. The search terms used in the systematic review will include behavioral therapy, behavior therapy, psychotherapy, and physical techniques. Chinese translations of these search terms will be used to search in Chinese databases. Search strategy for Medline is shown in Table 1.

**Table 1:**
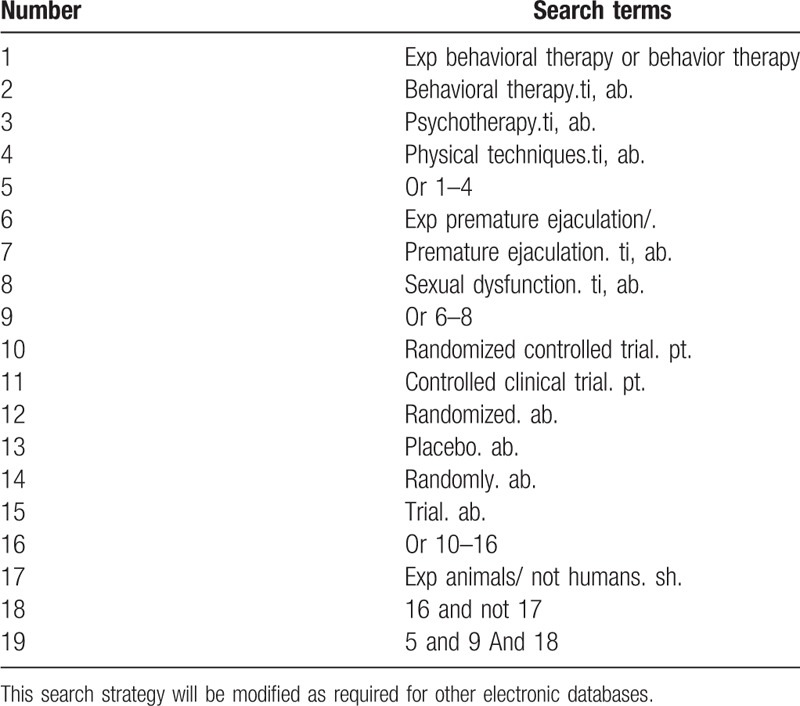
Search strategy used in PubMed database.

#### 2.3.2 Searching other resources

The reference lists of studies and systematic reviews related to premature ejaculation and behavioral therapy will be examined for additional trials. Ongoing trials which relevant to this term will be retrieved from the clinical registration platform, such as the WHO International Clinical Trials Registry Platform (ICTRP), the Chinese Clinical Trial Registry and ClinicalTrials.gov. We will manually search OpenGrey.eu for gray literature and ongoing trials in clinical trials.

### 2.4 Data collection and analysis

#### 2.4.1 Selection of studies

**Figure 1:**
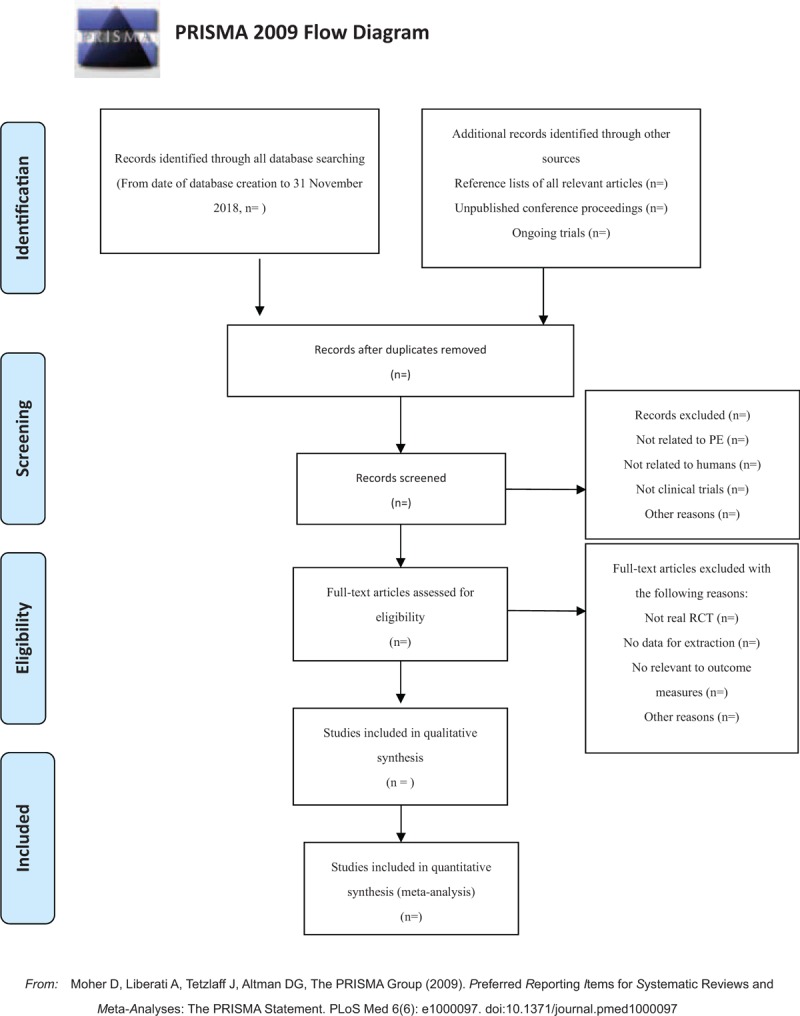
The PRISMA flow chart. PRISMA = Preferred Reporting Items for Systematic Reviews and Meta-Analyses.

Before the search begins, each reviewer will receive professional training to ensure consistency in the selection process and avoid the risk of bias (ROB) in human factors. The screening process will use EndNote X7 literature management software. Each literature of title and abstract is scanned by 2 reviewers (BHB and KGZ). All relevant articles of full text are investigated. When the 2 reviewers cannot reach a consensus on the selection process through consultations, the 3rd reviewer (JSW) will ultimately make the decision. The primary selection process is shown in a PRISMA flow chart (Fig. 1)

#### 2.4.2 Data extraction and management

Two reviewers (BHB and KGZ) will extract the necessary information for the systematic review from the included documents. This information will form a detail extraction form. If the details in the literature are incomplete, we will contact the author through via email, telephone, etc. The following data will be extracted:

- General information: research identification, publication time, title of article, correspondent author, contact information.
- Study methods: study design, sample size, randomization method, allocation concealment, blinding, incomplete report or selecting report, other sources of bias.
- Participants: inclusion criteria and exclusion criteria, age of patients, gender, PE diagnostic criteria, severity, race, research site, baseline of ejaculation time.
- Interventions: type of behavioral therapy, the dose of the medicine, treatment duration and frequency.
- Outcomes: primary, secondary, and safety outcomes as described above in the type of outcome measures part.
- Notes: financial support, conflicts of interest, ethical approval, important citations.

#### 2.4.3 Assessment of risk of bias in included studies

Risk of bias is used to evaluate the quality of study with the Cochrane Collaboration's risk-of-bias assessment method and complete the STRICTA checklist for the included studies. The decision of risk is made by 2 reviewers (JWS and XL). If there are inconsistent results appearing, the final decisions will be made by the 3rd author (BW). For missing or ambiguous data, we will try to contact the author as possible, and for duplicate publication we only select the original.

#### 2.4.4 Measures of treatment effect

For continuous data, if the results are measured on the same scale, we will calculate the mean difference (MD) and 95% confidence interval (CI). Standardized MDs with 95% CIs will be used if specific outcome metrics are measured using different outcome measurement scales. The relative risk (RR) will be used to evaluate the enumeration data.

#### 2.4.5 Unit of analysis issues

To avoid legacy effects, we will only extract the data of the 1st experimental period from the cross test. Through multiple intervention groups, we will combine all similar experimental groups and control groups into 1 to prevent analysis unit problems.

#### 2.4.6 Dealing with missing data

If there are missing or incomplete data for the primary results, we will attempt to get information by contacting the corresponding author of the referenced articles for the missing data. If the missing data cannot be obtained, we will perform our analysis based on the available data.

#### 2.4.7 Assessment of heterogeneity

We will use a Chi-square test to estimate heterogeneity of both the MD and OR. Further analysis can be performed using the I2 test. If possible, we will also construct a forest plot for analysis. A random-effect model will be used to interpret the results if heterogeneity is statistically significant, whereas a fixed-effect model will be used if heterogeneity is not statistically significant. We will regard heterogeneity as substantial when I2 is greater than 50% or a low *P* value (<.10) is reported for the Chi-square test for heterogeneity.^[16]^

#### 2.4.8 Assessment of reporting bias

If more than 10 trials are included in the meta-analysis, the visual asymmetry on the funnel plot will be used to evaluate the reported bias. If funnel plot asymmetry is detected, the reasons for this outcome will be analyzed.

#### 2.4.9 Data synthesis

RevManV.5.3.5 software will be used for all statistical analyses.^[17]^ We will decide to use a fixed effect model or a stochastic effect model based on the level of heterogeneity included in the study. If there was no statistical heterogeneity between the results, the fixed effect model will be used for meta-analysis. If considerable heterogeneity exists, we will use a 95% CIs stochastic effect model to analyze merge effect estimates.

#### 2.4.10 Subgroup analysis

If there is significant heterogeneity in the included trials, we will conduct a subgroup analysis based on the control intervention and different outcomes.

#### 2.4.11 Sensitivity analysis

We will conduct a sensitivity analysis to identify whether the conclusions are robust in the review according to the following criteria: sample size, heterogeneity qualities, and statistical model (random-effects or fixed-effects model).

#### 2.4.12 Grading the quality of evidence

We will evaluate the quality of evidence by the Grading of Recommendations Assessment, Development and Evaluation (GRADE) and will rate the quality by the following 4 levels: very low, low, moderate, and high.^[18]^

## 3 Discussion

As one of the most common sexual dysfunction diseases in males, the pathophysiology of PE has not been clear in clinical studies. Possible factors include anxiety and depression, penis allergy, 5-HT receptor dysfunction, and prostate inflammation, etc.^[19–21]^ In addition, the negative effects of PE go beyond sexual dysfunction. The PE can adversely affect a man's confidence, sometimes causing mental distress, anxiety, embarrassment, and depression that can seriously affect his normal life and his relationship with his sexual partner.^[22–23]^

Although PE can have serious psychological impact and could reduce the quality of life, few men seek treatment for it. In the Global Study of Sexual Attitudes and Behaviors survey, 78% of men who reported sexual dysfunction did not seek professional help or advice to address their sexual problems, however, the patients who suffer from erectile dysfunction (ED) are more likely to seek treatment.^[24]^

At present in the treatment of premature ejaculation, drug treatment as a 1st-line treatment. This includes short-acting dapoxetine (DPX) or other non-standard antidepressants on demand, such as the daily selective serotonin reuptake inhibitor (SSRIs). Tramadol and local anesthesia can be used as weak selection for SSRIs. Phosphodiesterase -5 inhibitors (PDE5is) are only available for men with ED.^[25]^

As a treatment with less side effect and lower cost, behavioral therapy aims to improve self-confidence and relieve anxiety and depression by continuously training men to master certain sexual skills to delay ejaculation time.

Including psychotherapy and physical techniques.^[26]^ Psychotherapy uses counseling to detect and correct any interpersonal problems that can lead to PE. Physical techniques includes ‘stop-start’, ‘squeeze’, ‘sensate focus’, and pelvic floor muscle rehabilitation.

Studies have shown that the short-term effect of behavioral therapy can reach 45% to 65%,^[27]^ but the long-term effect is still unclear. Increasing attention has been paid to behavioral therapy combined with other methods to treat PE. However, low-quality and small-sample results have been found in the present study.

We hope this review could provide more evidence. There are some limitations in this review. Due to language barriers, only studies published in English and Chinese will be included. Different types of behavioral therapy and degree of PE may run the risk of heterogeneity.

## Author contributions

**Data curation:** Binghao Bao, Kaige Zhang.

**Formal analysis:** Binghao Bao, Xiao Li.

**Funding acquisition:** haisong Li.

**Project administration:** Bin Wang.

**Supervision:** Jianwei Shang, Bin Wang.

**Validation:** haisong Li.

**Writing – original draft:** Binghao Bao, Jisheng Wang.

**Writing – review & editing:** Jianwei Shang, Hengheng Dai.
